# Short term outcomes and resource utilization in *de-novo* versus acute on chronic heart failure related cardiogenic shock: a nationwide analysis

**DOI:** 10.3389/fcvm.2024.1454884

**Published:** 2024-09-09

**Authors:** Mary Quien, Ju Young Bae, Sun-Joo Jang, Carlos Davila

**Affiliations:** ^1^ Section of Cardiovascular Medicine, Department of Internal Medicine, Yale New Haven Health Bridgeport Hospital, Bridgeport, CT, United States; ^2^ Section of Cardiovascular Medicine, Department of Internal Medicine, Yale New Haven Hospital, New Haven, CT, United States

**Keywords:** cardiogenic shock, *de novo* heart failure, critical care cardiology, chronic heart failure, advanced heart failure

## Abstract

**Background:**

There has been growing recognition of non-ischemic etiologies of cardiogenic shock (CS). To further understand this population, we aimed to investigate differences in clinical course between acute on chronic heart failure related (CHF-CS) and de-novo CS (DN-CS).

**Methods:**

Using the Nationwide Readmission Database, we examined 92,426 CS cases. Outcomes of interest included in-hospital and 30-day outcomes and use of advanced heart failure therapies.

**Results:**

Patients with DN-CS had higher in-hospital mortality than the CHF-CS cohort (32.6% vs. 30.4%, *p* < 0.001). Mechanical circulatory support (11.9% vs. 8.6%, *p* < 0.001) was more utilized in DN-CS. Renal replacement therapy (13.8% vs. 15.5%, *p* < 0.001) and right heart catheterization (16.0% vs. 21.0%, *p* < 0.001) were implemented more in the CHF-CS cohort. The CHF-CS cohort was also more likely to undergo LVAD implantation (0.4% vs. 3.6%, *p* < 0.001) and heart transplantation (0.5% vs. 2.0%, *p* < 0.001). Over the study period, advanced heart failure therapy utilization increased, but the proportion of patients receiving these interventions remained unchanged. Thirty days after index hospitalization, the CHF-CS cohort had more readmissions for heart failure (1.1% vs. 2.4%, *p* < 0.001) and all causes (14.1% vs. 21.1%, *p* < 0.001) with higher readmission mortality (1.1% vs. 2.3%, *p* < 0.001).

**Conclusion:**

Our findings align with existing research, demonstrating higher in-hospital mortality in the DN-CS subgroup. After the index hospitalization, however, the CHF-CS cohort performed worse with higher all-cause readmission rate and readmission mortality. The study also underscores the need for further investigation into the underutilization of certain interventions and the observed trends in the management of these CS subgroups.

## Introduction

Cardiogenic shock (CS) is defined as severe myocardial functional impairment resulting in systemic hypoperfusion, reduced cardiac output, and hypoxemia. Traditionally, research has focused on the pathophysiology and management of CS resulting from acute myocardial infarction (AMI) due to its high mortality (1). However, heart failure related cardiogenic shock has emerged as a significant contributor to CS cases (2). One multicenter analysis of cardiac intensive care units in North America revealed that more than half of cases were unrelated to AMI (3).

To further understand non-AMI cardiogenic shock, studies have aimed to investigate differences in clinical course between acute on chronic heart failure related cardiogenic shock (CHF-CS) and newly diagnosed or “*de-novo*” cardiogenic shock (DN-CS). However, there are inconsistent findings with some studies demonstrating higher in-hospital mortality in DN-CS while another suggesting overall worse long-term outcomes in the acute on chronic heart failure population (3–6). In addition, mortality in CS patients has not significantly improved over time, and the use of advanced heart failure resources remains variable across the country given the lack of evidence-based guidelines for resource use in these groups.

Using the Nationwide Readmission Database (NRD), we aim to compare the clinical course and outcomes in patients with DN-CS and CHF-CS.

## Method

This was a retrospective study using the data from the Nationwide Readmissions Database (NRD) between January 2016 and November 2019. The NRD is a large, publicly available, administrative database constructed using discharge data from Healthcare Cost and Utilization Project State Inpatient Databases in the United States. The NRD is developed through a Federal-State-Industry partnership sponsored by the Agency for Healthcare Research and Quality (AHRQ). Each record in the NRD contains information on the patient's diagnoses and procedures performed during the hospitalization, based on International Classification of Diseases, Tenth Revision-Clinical Modification (ICD-10-CM) codes or Procedure Coding System (ICD-10-PCS). We identified our study population, comorbidities, causes of readmissions and in-hospital outcomes using a combination of ICD-10-CM and ICD-10-PCS. Institutional Review Board approval and informed consent were not required for this study since all the data is de-identified and publicly available.

**Figure 1:**
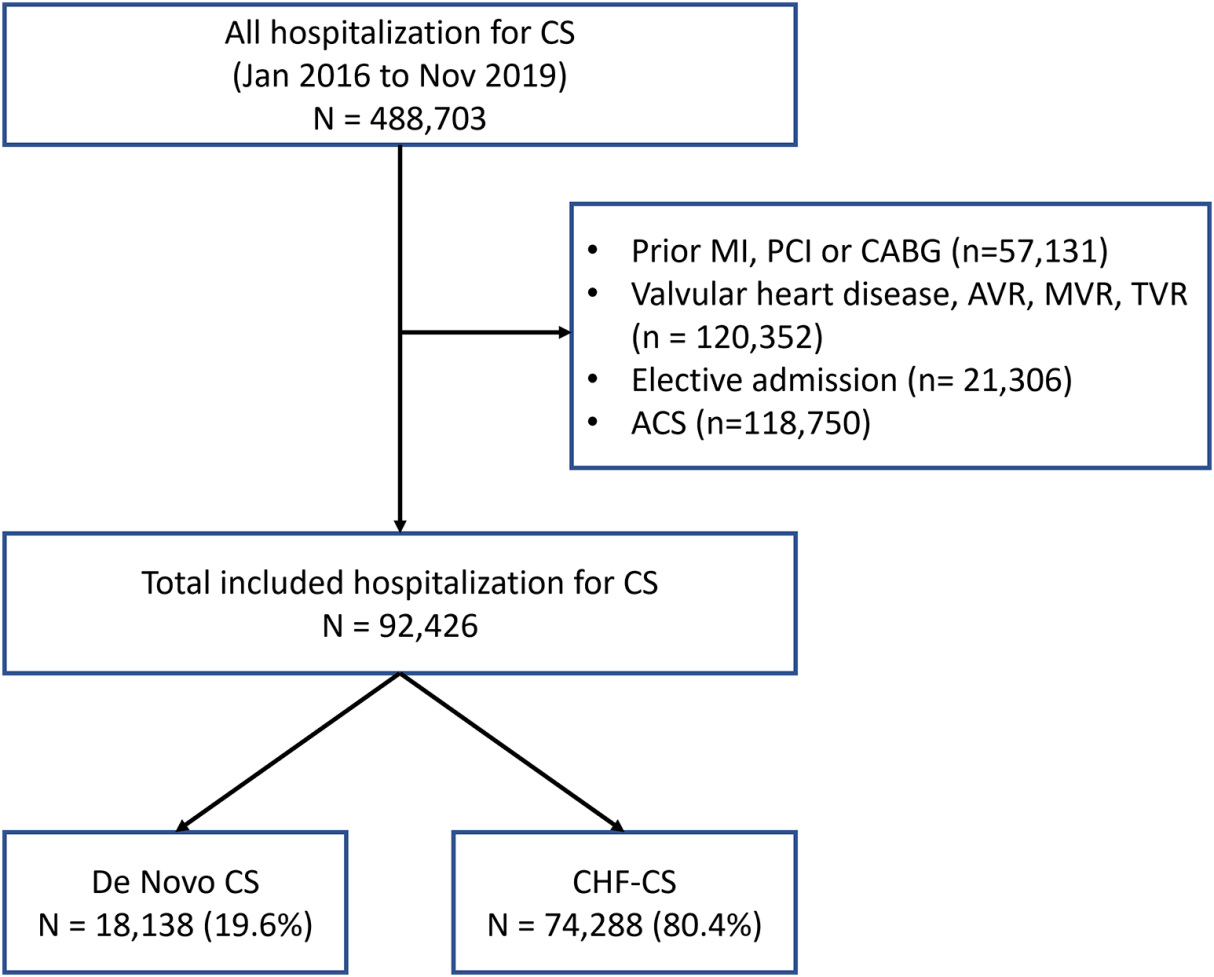
Flowchart of study population.

From January 2016 to November 2019, patients with cardiogenic shock were identified using ICD-10-CM codes for cardiogenic shock as the primary or secondary diagnosis (Figure 1). Patient variables were obtained from the database and included in our analysis as baseline characteristics. Since NRD prohibits linking patients across years, patients whose index hospitalization was in December were excluded in order to allow for completeness of data on thirty days of follow-up after discharge, similar to other prior studies examining the NRD (7–9). Patients were excluded if they carried a diagnosis of prior myocardial infarction, percutaneous coronary intervention, coronary artery bypass graft (CABG), or aortic valve replacement, tricuspid valve replacement, mitral valve replacement, or any valvular heart disease. Patients deemed to have an “elective admission” were also excluded. Patients were then divided into 2 groups: DN-CS or CHF-CS based on whether or not they carried a prior diagnosis of heart failure. The ICD codes used for the inclusion and exclusion criteria can be found in the Supplementary Material.

The primary outcome of interest was in-hospital outcomes and used of advanced heart failure therapies, over the course of 2016–2019. The secondary outcomes were thirty-day outcomes, excluding in-hospital death, after index hospitalization for cardiogenic shock. For thirty-day readmissions, only the first readmission within thirty days of the discharge was included. Transfer to another hospital was not included in the analysis since same-day readmissions and transfers are combined in a single variable in the NRD, and it does not specify if the transfer is to a higher or lower level of care.

All statistical analyses were performed using R statistical software, version 4.3.2, with its package “survey”. Discharge weight and stratum provided by NRD were used for all analyses and thus all the reported numbers are weighted national estimates. Domain analysis was used for accurate variance calculations for subgroup analyses. All analyses accounted for NRD sampling design by including hospital-year fixed effects based on hospital identification number. Categorical variables are presented as frequencies and analyzed by the Rao-Scott chi-square test. Continuous variables are shown as mean with standard error or median with interquartile range and are tested by either the Mann–Whitney–Wilcoxon test or a survey-specific linear regression test. All tests were two-sided with *p* value < 0.05 considered statistically significant.

## Results

Over the course of 2016–2019, there were 92,426 patients who presented with cardiogenic shock. 18,138 (19.6%) were determined to have DN-CS and 74,288 (80.4%) to have CHF-CS. The average age was 65 ± 0.1 years in the CHF-CS group and 62.6 ± 0.2 years (*p* < 0.001) in the DN-CS group. 56.3% of patients in the DN-CS group were male, compared to 63.6% of patients in the CHF-CS group. The CHF-CS group had significantly more comorbidities than the DN-CS group, including diabetes (31.2% vs. 42.0%, *p* < 0.001), pulmonary hypertension (12.8% vs. 24.0%, *p* < 0.001), chronic pulmonary disease (25.7% vs. 32.1%, *p* < 0.001), obesity (19.1% vs. 24.6%, *p* < 0.001), and chronic kidney disease (28.8% vs. 53.7%, *p* < 0.001). Further characteristics of each group are described in Table 1.

**Table 1:** Patient and hospital characteristics for patients hospitalized with cardiogenic shock.

Characteristics	Overall	DN-CS	CHF-CS	*P* value
Number of patients, *n* (%)	92,426	18,138 (19.6)	74,288 (80.4)	
Number of patients per year, *n* (%)				<0.001^a^
2016	16,632	3,532 (21.2)	13,100 (78.8)	
2017	19,077	3,650 (19.1)	15,427 (80.9)	
2018	26,227	5,293 (20.2)	20,934 (79.8)	
2019	30,489	5,663 (18.6)	24,826 (81.4)	
Patient characteristics, *n* (%)				
Age, mean (SE), year	63.9 (0.2)	61.9 (0.2)	64.4 (0.2)	<0.001^b^
Gender				<0.001
Male, *n* (%)	57,440 (62.1)	10,220 (56.3)	47,220 (63.6)	
Female, *n* (%)	34,986 (37.9)	7,918 (43.7)	27,068 (36.4)	
Smoking, *n* (%)	17,972 (19.4)	3,397 (18.7)	14,575 (19.6)	0.079
Hypertension, *n* (%)	11,399 (12.3)	2,948 (16.3)	8,451 (11.4)	<0.001
Diabetes mellitus, *n* (%)	36,899 (39.9)	5,661 (31.2)	31,238 (42.0)	<0.001
Dyslipidemia, *n* (%)	26,821 (29.0)	4,517 (24.9)	22,304 (30.0)	<0.001
Coronary artery disease, *n* (%)	23,941 (25.9)	3,553 (19.6)	20,388 (27.4)	<0.001
Peripheral vascular disease, *n* (%)	8,095 (8.8)	1,613 (8.9)	6,482 (8.7)	0.634
Previous stroke, *n* (%)	4,886 (5.3)	766 (4.2)	4,120 (5.5)	<0.001
Chronic pulmonary disease, *n* (%)	28,508 (30.8)	4,653 (25.7)	23,855 (32.1)	<0.001
Pulmonary hypertension, *n* (%)	20,186 (21.8)	2,327 (12.8)	17,859 (24.0)	<0.001
Pulmonary circulatory disorder, *n* (%)	20,983 (22.7)	2,442 (13.5)	18,541 (25.0)	<0.001
Chronic kidney disease, *n* (%)	45,133 (48.8)	5,226 (28.8)	39,907 (53.7)	<0.001
Liver disease, *n* (%)	14,463 (15.6)	2,357 (13.0)	12,106 (16.3)	<0.001
Atrial fibrillation, *n* (%)	42,721 (46.2)	6,841 (37.7)	35,880 (48.3)	<0.001
Obesity, *n* (%)	21,754 (23.5)	3,461 (19.1)	18,293 (24.6)	<0.001
Cerebrovascular disease, *n* (%)	7,183 (7.8)	1,485 (8.2)	5,698 (7.7)	<0.001
Dementia, *n* (%)	4,862 (5.3)	995 (5.5)	3,867 (5.2)	0.294
Depression, *n* (%)	9,291 (10.1)	1,817 (10.0)	7,474 (10.1)	0.912
Cardiac arrest, *n* (%)	5,707 (6.2)	1,532 (8.4)	4,175 (5.6)	<0.001
Length of hospital stay, d (IQR)	11 (6–19)	10 (5–17)	11 (6–20)	<0.001

DN-CS, *de novo* cardiogenic shock; CHF-CS, acute on chronic heart failure related cardiogenic shock; SE, standard error.

^a^ Rao-Scott *χ*^2^ test was used for all statistical tests unless stated otherwise.

^b^ Survey-specific linear regression was performed.

For in-hospital outcomes (Table 2), patients with DN-CS had overall higher in-hospital mortality than the CHF-CS cohort (32.6% vs. 30.4%, *p* < 0.001) and were more likely to present with cardiac arrest during the index hospitalization (8.4% vs. 5.6%, *p* < 0.001). In-hospital mortality was significantly higher in patients with DN-CS than patients with CHF-CS especially in 2019. Renal replacement therapy (RRT) (13.8% vs. 15.5%, *p* < 0.001) and right heart catheterization (RHC) (16.0% vs. 21.0%, *p* < 0.001) were implemented more in the CHF-CS cohort than in the DN-CS cohort, whereas mechanical circulatory support (MCS) (11.9% vs. 8.6%, *p* < 0.001) was more utilized in the DN-CS than in the CHF-CS group. The CHF-CS cohort was more likely to undergo LVAD implantation (0.4% vs. 3.6%, *p* < 0.001) and heart transplantation (0.5% vs. 2.3%, *p* < 0.001) than the DN-CS cohort. The absolute number of heart failure resources increased over time, but the proportion of patients who received these resources did not appear to significantly change.

**Table 2:** In-hospital outcomes for patients hospitalized with cardiogenic shock.

Characteristics	Overall (*n* = 92,426)	DN-CS (*n* = 18,138)	CHF-CS (*n* = 74,288)	*P* value
In-hospital mortality, *n* (%)	28,510 (30.8)	5,910 (32.6)	22,600 (30.4)	<0.001
2016	5,530/16,631 (33.3)	1,252/3,531 (35.5)	4,278/13,100 (32.7)	0.066
2017	6,106/19,077 (32.0%)	1,221/3,650 (33.5%)	4,885/15,427 (31.7%)	0.154
2018	8,116/26,227 (30.9%)	1,625/5,293 (30.7%)	6,491/20,934 (31.0%)	0.786
2019	8,756/30,489 (28.7%)	1,811/5,663 (32.0%)	6,945/24,826 (28.0%)	<0.001
Right heart catheterization, *n* (%)	18,467 (20.0%)	2,899 (16.0%)	15,568 (21.0%)	<0.001
2016	3,500/16,631 (21.0%)	616/3,531 (17.4%)	2,884/13,100 (22.0%)	<0.001
2017	3,860/19,077 (20.2%)	580/3,650 (15.9%)	3,280/15,427 (21.3%)	<0.001
2018	5,084/26,227 (19.4%)	825/5,293 (15.6%)	4,259/20,934 (20.3%)	<0.001
2019	6,022/30,489 (19.8%)	877/5,663 (15.5%)	5,145/24,826 (20.7%)	<0.001
Mechanical circulatory support, *n* (%)	8,531 (9.2%)	2,166 (11.9%)	6,365 (8.6%)	<0.001
2016	1,567/16,631 (9.4%)	387/3,531 (11.0%)	1,180/13,100 (9%)	0.060
2017	1,900/19,077 (10.0%)	452/3,650 (12.4%)	1,448/15,427 (9.4%)	<0.001
2018	2,354/26,227 (9.0%)	685/5,293 (12.9%)	1,669/20,934 (8.0%)	<0.001
2019	2,710/30,489 (8.9%)	643/5,663 (11.4%)	2,067/24,826 (8.3%)	<0.001
RRT, *n* (%)	14,030 (15.2)	2,508 (13.8)	11,522 (15.5)	<0.001
2016	2,306/16,631 (13.9%)	441/3,531 (12.5%)	1,865/13,100 (14.2%)	0.082
2017	2,805/19,077 (14.7%)	501/3,650 (13.%7)	2,304/15,427 (14.7%)	0.179
2018	4,217/26,227 (16.1%)	746/5,293 (14.1%)	3,471/20,934 (16.6%)	0.006
2019	4,703/30,489 (15.4%)	820/5,663 (14.5%)	3,883/24,826 (15.6%)	0.133
LVAD, *n* (%)	2,763 (3.0)	68 (0.4)	2,695 (3.6)	<0.001
2016	649/16,631 (3.9)	10/3,531 (0.3)	639/13,100 (4.9)	<0.001
2017	643/19,077 (3.4)	10/3,650 (0.3)	633/15,427 (4.1)	<0.001
2018	749/26,227 (2.9)	29/5,293 (0.5)	720/20,934 (3.4)	<0.001
2019	720/30,489 (2.4)	18/5,663 (0.3)	702/24,826 (2.8)	<0.001
Heart Transplantation, *n* (%)	1,783 (1.9)	92 (0.5)	1,691 (2.3)	<0.001
2016	306/16,631 (1.8)	27/3,531 (0.8)	279/13,100 (2.1)	0.014
2017	375/19,077 (2.0)	27/3,650 (0.7)	348/15,427 (2.3)	<0.001
2018	427/26,227 (1.6)	19/5,293 (0.4)	408/20,934 (1.9)	<0.001
2019	674/30,489 (2.2)	19/5,663 (0.3)	655/24,826 (2.6)	<0.001
Vascular complications, *n* (%)	1,233 (1.3)	283 (1.6)	950 (1.3)	0.040
2016	312/16,631 (1.9)	54/3,531 (1.5)	258/13,100 (2.0)	0.245
2017	272/19,077 (1.4)	84/3,650 (2.3)	188/15,427 (1.2)	0.002
2018	299/26,227 (1.1)	59/5,293 (1.1)	240/20,934 (1.1)	0.866
2019	352/30,489 (1.2)	87/5,663 (1.5)	265/24,826 (1.1)	0.032
Bleeding complications, *n* (%)	13,204 (14.3)	2,706 (14.9)	10,498 (14.1)	0.108
2016	238/166,312 (14.3)	545/3,531 (15.4)	1,837/13,100 (14.0)	0.338
2017	2,733/19,077 (14.3)	529/3,650 (14.5)	2,204/15,427 (14.3)	0.843
2018	3,691/26,227 (14.1)	790/5,293 (14.9)	2,901/20,934 (13.9)	0.018
2019	4,398/30,489 (14.4)	842/5,663 (14.9)	3,556/24,826 (14.3)	0.490

CHF-CS, acute on chronic heart failure related cardiogenic shock; DN-CS, *de novo* cardiogenic shock; IHM, invasive hemodynamic monitoring; LVAD, left ventricular assist device; RRT, renal replacement therapy.

Analysis of thirty days after index hospitalization (Table 3) revealed that the CHF-CS cohort had significantly higher incidence of readmissions for heart failure (1.1% vs. 2.4%, *p* < 0.001) and all causes (14.1% vs. 21.1%, *p* < 0.001) and higher readmission mortality (1.1% vs. 2.3%, *p* < 0.001) than the DN-CS cohort.

**Table 3:** Thirty-day outcomes (excluding in-hospital death) for patients hospitalized with cardiogenic shock.

Characteristics	Overall (*n* = 63,915)	DN-CS (*n* = 12,227)	CHF-CS (*n* = 51,688)	*P* value
All-cause readmission, *n* (%)	12,629 (19.8)	1,729 (14.1)	10,900 (21.1)	<0.001
Unplanned readmission, *n* (%)	12,035 (18.8)	1,615 (13.2)	10,420 (20.2)	<0.001
2016	2,203/11,101 (19.8)	342/2,279 (15.0)	1,861/8,822 (21.1)	<0.001
2017	2,434/12,970 (18.8)	316/2,428 (13.0)	2,118/10,542 (20.1)	<0.001
2018	3,320/18,112 (18.3)	462/3,669 (12.6)	2,858/14,443 (19.8)	<0.001
2019	4,079/21,733 (18.8)	495/3,851 (12.9)	3,584/17,882 (20.0)	<0.001
Readmission mortality, *n* (%)	1,336 (2.1)	133 (1.1)	1,203 (2.3)	<0.001
Readmission for HF, *n* (%)	1,396 (2.2)	138 (1.1)	1,258 (2.4)	<0.001

DN-CS, *de novo* cardiogenic shock; CHF-CS, acute on chronic heart failure related cardiogenic shock; HF, heart failure.

## Discussion

Using a large administrative database, we found key differences in the outcomes of patients presenting with CS with DN-CS and CHF-CS. Compared to the CHF-CS cohort, the DN-CS cohort had a worse in-hospital mortality rate. However, the CHF-CS cohort was found to have a significantly higher thirty-day readmission rate of any kind and a higher readmission mortality rate. Our findings overall align with those elucidated by other studies that investigated similar cohorts. First, we found that the CHF-CS cohort made up the majority of CS cases, comprising about 82% of total cases of CS every year. Although this is greater than the percentages quoted by the studies done by the Critical Care Cardiology Trials Network Registry and the Cardiogenic Shock Working Group registry, which was closer to 70%, we were unable to differentiate primary ventricular failure from other etiologies such as severe valvular disease, incessant arrhythmia, and tamponade, which may account for this difference (5, 6).

Similar to these studies, we also confirmed a higher in-hospital mortality rate with the DN-CS cohort. As proposed by the aforementioned studies, this could be attributed to a greater severity of shock at presentation due to lack of chronic adaptation to low flow states (5). Although the NRD is unable to provide specific clinical details, this idea is supported by the higher rate of cardiac arrest found in our DN-CS cohort as well as the higher utilization of MCS devices. Interestingly, after the index hospitalization, the CHF-CS cohort performed worse than the DN-CS cohort, with a higher all cause readmission rate, including heart failure, and readmission mortality. The CHF-CS cohort was overall a more debilitated group compared to the DN-CS cohort with a significantly higher number of comorbidities. This places the CHF-CS cohort at higher risk of persistent organ dysfunction and complications during and after the index hospitalization.

In terms of resource utilization, the CHF-CS cohort was more likely to undergo RRT, RHC, LVAD placement, and heart transplant compared to the DN-CS cohort. This difference is similar to other studies and likely multifactorial (5, 6). CHF-CS patients had more comorbidities that may have precluded them from MCS use but were more likely to have had prior evaluations for advanced therapies. The increased medical complexity of the CHF-CS cohort may have also prompted the increased use of RHC to garner more information, especially since this cohort had a higher prevalence of known pulmonary hypertension, suggesting a more complicated hemodynamic profile. Given the varying practices of RHC utilization at different institutions, the exact rationale for its use is difficult to pinpoint. Other studies have also shown that the DN-CS cohort may have greater multi-organ dysfunction in the acute setting that precludes them from consideration of LVAD or heart transplant (6).

Interestingly, despite an increase in heart failure therapies from 2016 to 2019, the proportion of CS patients who received these therapies appears largely unchanged. The lack of RHC utilization is most surprising since several studies have suggested that the use of RHC have led to improved outcomes and suggest possible mortality benefits (10, 11). Moreover, societies such as the European Society of Cardiology specifically have recommended RHC for patients with severe heart failure who are being evaluated for MCS or transplant (12). Further studies may be needed to discover why RHC are underutilized in the management of CS patients and whether some types of CS benefit from RHC more than others. The lack of increase in MCS does differ from a study that showed increases in temporary MCS use after the revised UNOS system took place in 2018 (13). However, the study showed this was mainly the case in transplant centers, which may not be reflected in our study that looks at all centers across the United States. It is also possible that there is simply a lack of available resources to accommodate the growing number of CS patients.

There are several limitations to this study. First, this study is limited by its retrospective design. Second, since data was extracted from an administrative database, we are unable to derive further detail from patient cases. As described above, this does not allow us to explore the clinical variables that may have played a role in the differences in outcomes between these two cohorts from other studies. Lastly, the NRD is only able to provide the occurrence of procedures but cannot relay the timing. The timing of RHC specifically has been shown to lead to shorter length of stay and lower readmission rates but needs to be further validated in future studies (11). The results from the study by Hernandez-Montfort and others also suggest that there is heterogeneity in the use and timing of MCS use in this patient population that needs further exploration (6).

This study underscores a paradox where, despite CHF-CS patients experiencing better in-hospital outcomes, their thirty-day results, including readmissions and readmission mortality, are worse. Our study brings attention to a high-risk population and possible underutilization of resources that could positively affect its clinical course.

## Data Availability

Publicly available datasets were analyzed in this study. This data can be found here: https://hcup-us.ahrq.gov/nrdoverview.jsp.

## References

[1] Thiele H Ohman EM de Waha-Thiele S Uwe Z Steffen D. Management of cardiogenic shock complicating myocardial infarction: an update 2019. Eur Heart J. (2019) 40(32):2671–83. 10.1093/eurheartj/ehz363 31274157

[2] Palacios Ordonez C Garan AR. The landscape of cardiogenic shock: epidemiology and current definitions. Curr Opin Cardiol. (2022) 37(3):236–40. 10.1097/HCO.0000000000000957 35275890

[3] Berg DD Bohula EA van Diepen S Katz JN Alviar CL Baird-Zars VM Epidemiology of shock in contemporary cardiac intensive care units. Circ Cardiovasc Qual Outcomes. (2019) 12(3):e005618. 10.1161/CIRCOUTCOMES.119.005618 30879324 PMC11032172

[4] Pranata R Tondas AE Yonas E Vania R Yamin M Chandra A Differences in clinical characteristics and outcome of *de novo* heart failure compared to acutely decompensated chronic heart failure—systematic review and meta-analysis. Acta Cardiol. (2021) 76(4):410–20. 10.1080/00015385.2020.1747178 32252602

[5] Bhatt AS Berg DD Bohula EA Alviar CL Baird-Zars VM Barnett CF *de novo* vs acute-on-chronic presentations of heart failure-related cardiogenic shock: insights from the critical care cardiology trials network registry. J Card Fail. (2021) 27(10):1073–81. 10.1016/j.cardfail.2021.08.014 34625127 PMC8514080

[6] Hernandez-Montfort J Kanwar M Sinha SS Garan AR Blumer V Kataria R Clinical presentation and in-hospital trajectory of heart failure and cardiogenic shock. JACC Heart Fail. (2023) 11(2):176–87. 10.1016/j.jchf.2022.10.002 36342421

[7] Jang SJ Kim LK Sobti NK Yeo I Cheung JW Feldman DN Mortality of patients with ST-segment-elevation myocardial infarction without standard modifiable risk factors among patients without known coronary artery disease: age-stratified and sex-related analysis from nationwide readmissions database 2010–2014. Am J Prev Cardiol. (2023) 14:100474. 10.1016/j.ajpc.2023.100474 36923367 PMC10009437

[8] Jang SJ Yeo I Jonas C Goyal P Cheung JW Feldman DN Thirty-day readmission rates after takotsubo syndrome with or without malignancy: a nationwide readmissions database analysis. J Clin Med. (2021) 10(16):3701. 10.3390/jcm10163701 34441995 PMC8397058

[9] Jang SJ Yeo I Feldman DN Cheung JW Minutello RM Singh HS Associations between hospital length of stay, 30-day readmission, and costs in ST-segment-elevation myocardial infarction after primary percutaneous coronary intervention: a nationwide readmissions database analysis. J Am Heart Assoc. (2020) 9(11):e015503. 10.1161/JAHA.119.015503 32468933 PMC7428974

[10] Ranka S Mastoris I Kapur NK Tedford RJ Rali A Acharya P Right heart catheterization in cardiogenic shock is associated with improved outcomes: insights from the nationwide readmissions database. J Am Heart Assoc. (2021) 10(17):e019843. 10.1161/JAHA.120.019843 34423652 PMC8649238

[11] Elzanaty AM Maraey A Khalil M Elsharnoby H Nazir S Moukarbel GV. Right heart catheterization timing and outcomes of cardiogenic shock: analysis from the national readmission database. Curr Probl Cardiol. (2022) 47(12):101388. 10.1016/j.cpcardiol.2022.101388 36058343

[12] McDonagh TA Metra M Adamo M Gardner RS Baumbach A Bohm M 2021 ESC guidelines for the diagnosis and treatment of acute and chronic heart failure [published correction appears in Eur Heart J. 2021 Oct 14;:]. Eur Heart J. (2021) 42(36):3599–726. 10.1093/eurheartj/ehab368 34447992

[13] Berg DD Barnett CF Kenigsberg BB Kenigsberg BB Papolos A Alviar CL Clinical practice patterns in temporary mechanical circulatory support for shock in the critical care cardiology trials network (CCCTN) registry. Circ Heart Fail. (2019) 12(11):e006635. 10.1161/CIRCHEARTFAILURE.119.006635 31707801 PMC7008928

