# Supplementary material for: Short term outcomes and resource utilization in de-novo versus acute on chronic heart failure related cardiogenic shock: a nationwide analysis
Source: Front Cardiovasc Med. 2024 Sep 9;11:1454884. doi: 10.3389/fcvm.2024.1454884 (PMC11416976; doi:10.3389/fcvm.2024.1454884)
Supplement: Supplementary file 1 [file Table1.docx]

Supplementary Table A. International Classification of Diseases, Tenth Revision-Clinical Modification (ICD-10-CM) codes and Procedure Coding System (ICD-10-PCS) for selected variables.

| Variables: | ICD-10-CM or ICD-10-PCS codes |
| --- | --- |
| Cardiogenic shock | "R570" |
| Acute heart failure | "I5020","I5021","I5030","I5031","I5041" |
| Chronic heart failure | "I50813","I5023","I5032","I5033","I5042","I5043", "I50812", "I50813" |
| Prior myocardial infarction | "I252" |
| Prior percutaneous coronary intervention | "Z955" |
| Prior coronary artery bypass graft | "Z951" |
| TAVR | "02RF37H", "02RF37Z", "02RF38H", "02RF38Z", "02RF3JH", "02RF3JZ", "02RF3KH", "02RF3KZ" |
| SAVR | "02RF07Z", "02RF08Z", "02RF0JZ", "02RF0KZ" |
| Mitral valve replacement | "02RG07Z", "02RG08Z", "02RG0JZ", "02RG0KZ","02RG37H", "02RG37Z","02RG38H","02RG38Z", "02RG3JH", "02RG3JZ", "02RG3KH","02RG3KZ","02RG47Z","02RG48Z","02RG4JZ", "02RG4KZ" |
| Tricuspid valve replacement | 02RJ07Z", "02RJ08Z", "02RJ0JZ", "02RJ0KZ","02RJ37H", "02RJ37Z","02RJ38H","02RJ38Z","02RJ3JH","02RJ3JZ", "02RJ3KH","02RJ3KZ","02RJ47Z","02RJ48Z","02RJ4JZ","02RJ4KZ" |
| Acute coronary syndrome | "I2101","I2102","I2109","I2111","I2119","I2121","I220","I221","I222", "I228", "I229","I219","I213" |
| Cardiac arrest | "I469" |
| Intraaortic balloon pump | "5A02110","5A02210" |
| Percutaneous coaxial LVAD | "5A0221D","5A0211D","5A02116","5A02216" |
| ECMO | "5A1522F", "5A1522G", "5A15A2F", "5A15A2G", "5A15223" |
| LVAD | "02HA0QZ" |
| Heart transplantation | "02YA0Z0", "02YA0Z1" |
| Renal replacement therapy | "5A1D70Z", "5A1D80Z", "5A1D90Z", "5A1D60Z", "5A1D00Z" |
| Vascular complications | "S36899A","T81718A","T81719A","T8172XA","T81710A", "T81711A","T801XXA", "I770","S2500XA", "S2501XA", "S2502XA", "S2509XA","S3500XA","S3501XA","S3502XA", "S3509XA", "S75011A","S75012A","S75019A","S75021A","S75022A","S75029A", "S75099A","I97410","I97411","I97418", "I9742","I97610", "I97611", "I97618","I97620","L7602", "L7622","M96811","M96831","I9751",  "I9752","L7612","M96821","T8171","T8172","S25499A", "S3559XA", "S45001A","S45099A","S75001A", "S75199A","S85001A","S85599A" |
| Bleeding complications | "I97410","I97418","I97610","I97618","I97630","I9742","I97620", "I97410","I97411", "I97418","I9742","I97610","I97621","I97630", "I97640","I97618","I97621","L7602", "L7622","M9681","M9683", "M96811","K661","D62" |

Abbreviations: ECMO, extracorporeal membrane oxygenation; LVAD, left ventricular assist device; SAVR, surgical aortic valve replacement; TAVR, transcatheter aortic valve replacement
